# Some Inequalities Combining Rough and Random Information

**DOI:** 10.3390/e20030211

**Published:** 2018-03-20

**Authors:** Yujie Gu, Qianyu Zhang, Liying Yu

**Affiliations:** School of Management, Shanghai University, Shanghai 200444, China

**Keywords:** rough random variable, inequalities, critical values

## Abstract

Rough random theory, generally applied to statistics, decision-making, and so on, is an extension of rough set theory and probability theory, in which a rough random variable is described as a random variable taking “rough variable” values. In order to extend and enrich the research area of rough random theory, in this paper, the well-known probabilistic inequalities (Markov inequality, Chebyshev inequality, Holder’s inequality, Minkowski inequality and Jensen’s inequality) are proven for rough random variables, which gives a firm theoretical support to the further development of rough random theory. Besides, considering that the critical values always act as a vital tool in engineering, science and other application fields, some significant properties of the critical values of rough random variables involving the continuity and the monotonicity are investigated deeply to provide a novel analytical approach for dealing with the rough random optimization problems.

## 1. Introduction

Rough set theory, initially introduced by Pawlak [1] in 1982 as a mathematical tool to handle problems with insufficient information, has been tested and verified to be a remarkable instrument for dealing with uncertain situations. Its main assumption is that any object from a universe can be perceived through available information, and such information may not be sufficient to characterize the object exactly. Due to the fact that a splendid way of approximating a set is to use other sets, the two crisp sets, named as lower and upper approximations, are generally used to represent rough sets. So far, rough set theory has been applied to various fields especially data mining and biological information processing (see, e.g., [2,3,4,5]). Considering that a rough variable is of great significance to the optimization problems with rough information, Liu [6] in 2002 initiated a definition for a rough variable, that is a function converting the rough space to the real set. Based on the definition, some fundamental arithmetics, numerical characteristics, including the expectation value and variance, and other properties of rough variables are investigated and proven in succession. By virtue of the theory of rough variables developed by Liu [6], a great deal of problems in practical applications can be solved admirably (see, e.g., [7,8]).

It is known that uncertainties always exist of diverse types on account of the extreme complexity of the real world, some of which are possibly hard to describe by extant distinct theories. Hence, researchers turned to hybrid uncertainty theories, which indeed represent the problems in complex uncertain situations well, such as data reduction [9], neural networks [10], and so on. For example, serving as mathematical descriptions for fuzzy stochastic phenomena, the notion of a fuzzy random variable was introduced by Kwakernaak [11] first and then developed by several researchers such as Puri and Ralescu [12] and Kruse and Meyer [13] according to different requirements of measurability. Further, Liu [6] initially presented specific definitions for fuzzy rough variables, random fuzzy variables and bifuzzy variables and dived into the expectation, variance and other properties of them. By now, these hybrid uncertain variables have been diffusely applied to various fields, such as risk decision [14] and water management [15].

Among all these hybrid uncertainty theories, rough random theory is authoritative in managing the indeterministic problems that cannot be solved by rough set theory and probability theory separately. In 1990, Dubois and Prade [16] first introduced the rough random set, which is described as an approximation to a random set by a pair of upper and lower approximations. For example, in real life, a random set takes place with a certain probability in a random event in which the random set cannot be represented exactly, but its upper and lower approximate sets can be given. Then, the pair of sets are called rough random sets. Similar to the rough random set, the notion of a rough random variable was presented and well defined by Liu [6] in 2002 as a function from a probability space to the set of rough variables, or a random variable taking “rough variable” values. Following that, Liu [17] further discussed the chance measure, the chance distribution and some critical numerical characteristics of rough random variables. Subsequently, several sequel studies focused on the applications of rough random variables. For example, Wang et al. [18] employed rough random variables to guarantee the efficiency and accuracy of the final ranking of alternatives in decision making. Moreover, on account of improving the scheduling efficiency in the Pubugou Hydropower Project, rough random variables were introduced by Zhang and Xu [19] to the resource-constrained project scheduling problem.

As is well known, there are several proverbial inequalities in probability theory, including Markov inequality, Chebyshev inequality, Jensen’s inequality, Holder’s inequality and Minkowski inequality, based on which some critical properties of random variables involving laws of large numbers can be proven immediately, which immensely boosts the development of probability theory. In view of the significance of these inequalities, Liu [20] applied them to rough variables and fuzzy variables and then deduced many superior properties such as convergence in trust and convergence in the mean. Later, subsequent research intensively studied these inequalities further and concretely proved that these inequalities also hold for fuzzy random variables [21], fuzzy rough variables [22] and random fuzzy variables [23], which gives strong support to subsequent development of these two theories. Considering that it is of great significance in rough random theory, this paper proves these inequalities for rough random variables and also dives into the properties of their critical values.

The rest of this paper is organized as follows. In Section 2, an overview of the basic knowledge of random variables, rough variables and rough random variables is given including the chance measure and the expected value operator. Some inequalities for rough random variables are described and verified in Section 3. Based on the definitions of the (γ,δ)-optimistic value and the (γ,δ)-pessimistic value, Section 4 studies the monotonicity and continuity of the critical values. Finally, the conclusions are presented in Section 5.

## 2. Preliminaries

Roughly speaking, rough random variables are combinations of rough variables and random variables. Thus, in this section, some theoretical fundamentals of random variables, rough variables and rough random variables are retrospected to provide knowledge and theoretic preparation for the following sections.

### 2.1. Random Variable

Probability theory, initialized early in the 17th Century, immensely boosted the development of science, management, and so on. As a ripe theoretical system, the fundamental concepts in probability theory have been defined in a unified and standardized manner, some of which are recalled as follows.

**Definition** **1.** *Assume that* Ω *represents a nonempty set, A is a σ-algebra of subsets of* Ω *and Pr is a set function called a probability measure satisfying the following axioms:*

Axiom 1. Pr{∅}=0;

Axiom 2. Pr{Ω}=1;

Axiom 3. For any countable sequence of mutually-exclusive random events $A_{n}\in A$, n=1,2,⋯, we have $\mathrm{Pr}\left\{{⋃}_{n}A_{n}\right\}=\sum_{n}\mathrm{Pr}\left\{A_{n}\right\}$.

Then, (Ω,A,Pr) is called a probability space.

**Definition** **2.** Assume that a probability space is described by (Ω,A,Pr). Then, a random variable κ on the space is defined as a measurable function from the probability space to the real line R.

**Definition** **3.** *Assume that a random variable is represented by κ. Then, the probability distribution Φ(x) of κ represents the probability that the value of κ is no larger than a real number x, that is,*

(1) Φ(x)=Pr{τ∈Ω|κ(τ)≤x}.

**Definition** **4.** *Assume that a probability space is described by (Ω,A,Pr), and κ is a random variable on the space. Then, the expected value of κ, $E^{p}\left[\kappa \right]$, is defined by:*

(2) $$E^{p}\left[\kappa \right]=\int_{0}^{+\infty }\mathrm{Pr}\left\{\kappa \geq \rho \right\}d\rho -\int_{-\infty }^{0}\mathrm{Pr}\left\{\kappa \leq \rho \right\}d\rho ,$$

*provided that at least one of the two integrals is finite.*

**Theorem** **1.** *Assume that a probability space is described by (Ω,A,Pr), and κ and λ are two random variables with expected values $E^{p}\left[\kappa \right]$ and $E^{p}\left[\lambda \right]$, respectively. Then, we have:*

(3) $$E^{p}\left[i\kappa +j\lambda \right]=iE^{p}\left[\kappa \right]+jE^{p}\left[\lambda \right],$$

*where i and j are two real numbers.*

### 2.2. Rough Variable

As an important part of rough set theory, a rough variable is indeed a terrific tool to assist rough set theory in dealing with uncertain situations. For the sake of fully depicting rough variables, some related definitions were given by Liu [6] to handle the problems in rough set theory.

**Definition** **5.** *(Liu [6]) Assume that a set* Λ *is non-empty, A is a σ-algebra of subsets of* Λ *with element Δ∈A, and the set function π (a measure) satisfies three properties, i.e., real-valued, nonnegative and countable additive. Then,* (Λ, Δ, *A, π) is called a rough space.*

**Definition** **6.** *(Liu [17]) Assume that a rough space is described as* (Λ, Δ, *A, π). Then, the upper and lower trusts of an event A are defined as:*

(4) $$T\bar{r}\left\{A\right\}=\frac{\pi \{A\}}{\pi (\Lambda )},$$

(5) $$T\underline{r}\left\{A\right\}=\frac{\pi \{A\cap \Delta \}}{\pi (\Delta )},$$

*respectively. Then, the trust of the rough event A is defined by:*

(6) $$\mathrm{Tr}\left\{A\right\}=\frac{1}{2}\left(T\bar{r}\left\{A\right\}+T\underline{r}\left\{A\right\}\right).$$

**Definition** **7.** *(Liu [17]) Assume that a rough space is described as* (Λ, Δ, *A, π). Then, a rough variable κ is a measurable function from the rough space to the set of real numbers. That is, for every Borel set B of R, we have:*

(7) {τ∈Λ|κ(τ)∈B}∈A.

*The lower and the upper approximations of the rough variable κ are then defined as:*

(8) $$\underline{\kappa }=\left\{\kappa \left(\tau \right)|\tau \in \Delta \right\},\;\;\;\;\bar{\kappa }=\left\{\kappa \left(\tau \right)|\tau \in \Lambda \right\}.$$

**Example** **1.** *Assume that a rough space is described as* (Λ, Δ, *A, π), where Λ={o≤τ≤l}, Δ={p≤τ≤q} and o≤p<q≤l. Here, π is a length measure for a set; for example, if a set A=[a,b], then π{A}=b−a. A rough variable κ is defined on this space as an identity function κ(τ)=τ. Then, we can calculate the lower and upper trusts, as well as the trust of the event κ≤0, as follows:*

(9) $$T\bar{r}\left\{\kappa \leq 0\right\}=\left\{\begin{array}{cc} 0, & if\;o\geq 0\\ \frac{o}{o-l}, & if\;o\leq 0\leq l\\ 1, & if\;l\leq 0, \end{array}\right.$$

(10) $$T\underline{r}\left\{\kappa \leq 0\right\}=\left\{\begin{array}{cc} 0, & if\;p\geq 0\\ \frac{p}{p-q},\;\;\;\; & if\;p\leq 0\leq q\\ 1, & if\;q\leq 0, \end{array}\right.$$

(11) $$\mathrm{Tr}\left\{\kappa \leq 0\right\}=\left\{\begin{array}{cc} 0, & if\;o\geq 0\\ \frac{o}{2(o-l)}, & if\;o\leq 0\leq p\\ \frac{2op-pl-oq}{2(l-o)(q-p)}, & if\;p\leq 0\leq q\\ \frac{l-2o}{2(l-o)}, & if\;q\leq 0\leq l\\ 1, & if\;l\leq 0. \end{array}\right.$$

*Moreover, for the special case [p,q]=[o,l], we have Λ=Δ and $T\underline{r}\left\{\kappa \leq 0\right\}=T\bar{r}\left\{\kappa \leq 0\right\}$, and then, the trust Tr{κ≤0} becomes:*

(12) $$\mathrm{Tr}\left\{\kappa \leq 0\right\}=\left\{\begin{array}{cc} 0, & if\;p\geq 0\\ \frac{p}{p-q}, & if\;p\leq 0\leq q\\ 1, & if\;q\leq 0. \end{array}\right.$$

**Definition** **8.** *(Liu [6]) Assume that a rough space is described as* (Λ, Δ, *A, π) with a rough variable κ on this space. Then, the expected value of κ, $E^{r}\left[\kappa \right]$, is defined by:*

(13) $$E^{r}\left[\kappa \right]=\int_{0}^{+\infty }\mathrm{Tr}\left\{\kappa \geq \rho \right\}d\rho -\int_{-\infty }^{0}\mathrm{Tr}\left\{\kappa \leq \rho \right\}d\rho ,$$

**Example** **2.** *Assume that κ is a rough variable represented laconically by κ=([p,q],[o,l]) with o≤p<q≤l. Then, we have:*

(14) $$E^{r}\left[\kappa \right]=\frac{1}{4}\left(p+q+o+l\right).$$

*When p=o and q=l, we obtain:*

(15) $$E^{r}\left[\kappa \right]=\frac{1}{2}\left(q+p\right).$$

**Theorem** **2.** *(Liu [6]) Assume that a rough space is described by* (Λ, Δ, *A, π), κ and λ are two rough variables with expected values $E^{r}\left[\kappa \right]$ and $E^{r}\left[\lambda \right]$, respectively. Then, we have:*

(16) $$E^{r}\left[i\kappa +j\lambda \right]=iE^{r}\left[\kappa \right]+jE^{r}\left[\lambda \right],$$

*where i and j are two real numbers.*

### 2.3. Rough Random Variable

As a natural extension of rough set theory and probability theory, rough random theory was proposed by Liu [6] for studying the indeterminate phenomenon with combined rough and random information. In this section, for the purpose of describing a rough random variable more precisely, some elementary concepts of rough random variables, including the chance measure and expected value operator, are reviewed below.

**Definition** **9.** (Liu [17]) Assume that a probability space is described by (Ω,A,Pr). Then, a rough random variable κ on the space is defined as a function from the space to the set of rough variables such that Tr{κ(τ)∈O} is a measurable function of τ for any Borel set O of R.

**Definition** **10.** *(Liu [17]) Assume that a probability space is described by (Ω,A,Pr), κ is a rough random variable and $E^{r}$ represents the expected operator of rough variables. Then, the expected value of κ, E[κ], is defined by:*

(17) $$E\left[\kappa \right]=\int_{0}^{+\infty }\mathrm{Pr}\left\{\tau \in \Omega |E^{r}\left[\kappa \left(\tau \right)\right]\geq \rho \right\}d\rho -\int_{-\infty }^{0}\mathrm{Pr}\left\{\tau \in \Omega |E^{r}\left[\kappa \left(\tau \right)\right]\leq \rho \right\}d\rho ,$$

*provided that at least one of the two integrals is finite.*

**Theorem** **3.** *(Liu [17]) Assume that a probability space is described by (Ω,A,Pr) and κ and λ are two rough random variables with expected values E[κ] and E[λ], respectively. Then, we have:*

(18) E[iκ+jλ]=iE[κ]+jE[λ],

*where i and j are two real numbers.*

**Definition** **11.** *(Liu [17]) Assume that a probability space is described by (Ω,A,Pr) and κ is a rough random variable with expected value E(κ). Then, the variance of κ, V[κ], is defined by:*

(19) $$V\left[\kappa \right]=E[{\left(\kappa -E\left(\kappa \right)\right)}^{2}].$$

**Definition** **12.** *(Liu [17]) Assume that a probability space is described by (Ω,A,Pr) with a rough random variable κ on the space. Then, the chance of the event κ∈O, Ch{κ∈O}, is defined by:*

(20) $$\mathrm{Ch}\left\{\kappa \in O\right\}\left(\alpha \right)=\underset{\mathrm{Pr}\{A\}\geq \alpha }{\sup}\underset{\tau \in A}{\inf}\mathrm{Tr}\left\{\kappa \left(\tau \right)\in O\right\},$$

*where O is a Borel set of R.*

## 3. Inequalities of Rough Random Variables

In this section, the five celebrated inequalities (Markov inequality, Chebyshev inequality, Holder’s inequality, Minkowski inequality and Jensen’s inequality) commonly used in probability theory and demonstrated for rough variables by Liu [20] are also clarified for rough random variables, which will give substantial theoretical support for practical applications, involving decision making, resource-constrained project schedule, and so on, and then will make them well solved.

**Theorem** **4.** *Assume that a probability space is described by (Ω,A,Pr) with a rough random variable κ on the space. Provided that a function g is measurable, nonnegative and strictly increasing on [0,∞), then we have:*

(21) $$\mathrm{Ch}\left\{|\kappa |\geq r\right\}\left(\alpha \right)\leq \frac{E[g(\kappa )]}{\alpha g(r)},$$

*where r and α are two predetermined real numbers satisfying r>0 and α∈(0,1].*

**Proof of Theorem** **4.** Since κ(τ) is a rough variable where τ∈Ω, it follows from the inequality proven by Liu [17] that:

$$\mathrm{Tr}\left\{|\kappa \left(\tau \right)|\geq r\right\}\leq \frac{E[g(\kappa (\tau ))]}{g(r)}$$

for each τ. Specifically, when Pr{A}≥α where A⊂Ω, we have:

$$\underset{\tau^{*}\in A}{\inf}\mathrm{Tr}\{|\kappa \left(\tau^{*}\right)\left|\geq r\right\}\leq \frac{E[g(\kappa (\tau ))]}{g(r)},\;\forall \tau \in A.$$

Since the function *g* is nonnegative, we have:

$$\begin{array}{cc} \frac{E[g(\kappa )]}{g(r)}\;\;\;\; & =\frac{1}{g(r)}\int_{0}^{+\infty }\mathrm{Pr}\left\{\tau \in \Omega |E^{r}\left[g\left(\kappa \left(\tau \right)\right)\right]\geq \rho \right\}d\rho \\ & \geq \frac{1}{g(r)}\int_{0}^{+\infty }\mathrm{Pr}\left\{\tau \in A|E^{r}\left[g\left(\kappa \left(\tau \right)\right)\right]\geq \rho \right\}d\rho \\ & \geq \int_{0}^{+\infty }\mathrm{Pr}\{\tau \in A|\underset{\tau^{*}\in A}{\inf}\mathrm{Tr}\{|\kappa \left(\tau^{*}\right)|\geq r\}\geq \rho \}d\rho \\ & =\underset{\tau^{*}\in A}{\inf}\mathrm{Tr}\{|\kappa \left(\tau^{*}\right)\left|\geq r\right\}\cdot \mathrm{Pr}\left\{A\right\}\\ & \geq \underset{\tau^{*}\in A}{\inf}\mathrm{Tr}\{|\kappa \left(\tau^{*}\right)\left|\geq r\right\}\cdot \alpha . \end{array}$$

Simplifying the inequality, we obtain:

$$\underset{\tau^{*}\in A}{\inf}\mathrm{Tr}\{|\kappa \left(\tau^{*}\right)\left|\geq r\right\}\leq \frac{E[g(\kappa )]}{\alpha g(r)}.$$

Due to the arbitrariness of *A*, the above inequality can be rewritten as:

$$\underset{\mathrm{Pr}\{A\}\geq \alpha }{\sup}\underset{\tau^{*}\in A}{\inf}\mathrm{Tr}\{|\kappa \left(\tau^{*}\right)\left|\geq r\right\}\leq \frac{E[g(\kappa )]}{\alpha g(r)}.$$

That is,

$$\mathrm{Ch}\left\{|\kappa |\geq r\right\}\left(\alpha \right)\leq \frac{E[g(\kappa )]}{\alpha g(r)}.$$

  ☐

Especially, when the function *g* in Theorem 4 takes designated forms, the inequality (21) can be seen as the two well-known inequalities, Markov inequality and Chebyshev inequality, in rough random theory. A brief proof will be given in the following.

**Theorem** **5.** *(Markov inequality) Assume that a probability space is described by (Ω,A,Pr) with a rough random variable κ on the space. Then, we have:*

(22) $$\mathrm{Ch}\left\{|\kappa |\geq r\right\}\left(\alpha \right)\leq \frac{E[|\kappa {|}^{s}]}{\alpha r^{s}},$$

*where r, s and α are three predetermined real numbers satisfying r>0, s>0 and α∈(0,1].*

**Proof of Theorem** **5.** Suppose that the function g(x) is equal to ${\left|x\right|}^{s}$ with s>0. Then, for a positive number *r*, we have:

$$g\left(\kappa \right)={\left|\kappa \right|}^{s},\;g\left(r\right)={\left|r\right|}^{s}.$$

Substituting them into the inequality (21), then we have:

$$\mathrm{Ch}\left\{|\kappa |\geq r\right\}\left(\alpha \right)\leq \frac{E[g(\kappa )]}{\alpha g(r)}=\frac{E[|\kappa {|}^{s}]}{\alpha r^{s}}.$$

  ☐

**Theorem** **6.** *(Chebyshev inequality) Assume that a probability space is described by (Ω,A,Pr) and κ is a rough random variable with finite variance V[κ]. Then, we have:*

(23) $$\mathrm{Ch}\left\{|\kappa -E[\kappa ]|\geq r\right\}\left(\alpha \right)\leq \frac{V[\kappa ]}{\alpha r^{2}},$$

*where r and α are two predetermined real numbers satisfying r>0 and α∈(0,1].*

**Proof of Theorem** **6.** Based on Definition 11, the inequality (23) can be reformed as:

$$\mathrm{Ch}\left\{|\kappa -E\left[\kappa \right]|\geq r\right\}\left(\alpha \right)\leq \frac{E[{\left(\kappa -E\left(\kappa \right)\right)}^{2}]}{\alpha r^{2}}.$$

Letting κ=κ−E[κ], then we obtain:

$$\mathrm{Ch}\left\{|\kappa |\geq r\right\}\left(\alpha \right)\leq \frac{E[|\kappa {|}^{2}]}{\alpha r^{2}}.$$

In light of the Markov inequality, the inequality (23) is obtained immediately. ☐

Chebyshev and Markov inequalities describe the upper bound of chance with different conditions where the absolute value of a rough random variable is no less than a positive number, which state the fundamental properties of a rough random variable. Based on these, numerous inequalities about rough random variables can be verified with a slight alteration.

**Theorem** **7.** *Assume that a probability space is described by (Ω,A,Pr) and κ is a rough random variable with a finite expected value of its modulus $\tilde{e}$. Then, we have:*

(24) $$\mathrm{Ch}\{|\kappa |\geq \tilde{e}\}\left(\alpha \right)\leq \frac{1}{\alpha }.$$

**Proof of Theorem** **7.** Owing to κ being a rough random variable, it follows from Equation (22) that:

$$\mathrm{Ch}\left\{|\kappa |\geq r\right\}\left(\alpha \right)\leq \frac{E[|\kappa {|}^{s}]}{\alpha r^{s}},$$

where r>0 and s>0. Assuming that $r=\tilde{e}$ and s=1, respectively, it can be derived that:

$$\mathrm{Ch}\{|\kappa |\geq \tilde{e}\}\left(\alpha \right)\leq \frac{\tilde{e}}{\alpha \tilde{e}}=\frac{1}{\alpha }.$$

  ☐

**Theorem** **8.** *(Holder’s inequality) Assume that a probability space is described by (Ω,A,Pr) and κ and λ are two rough random variables on this space. Provided that s and t are two positive numbers satisfying 1/s+1/t=1, then we have:*

(25) $$E\left[|\kappa \lambda |\right]\leq \sqrt[{s}]{E[|\kappa {|}^{s}]}\cdot \sqrt[{t}]{E[|\lambda {|}^{t}]},$$

*where $E[|\kappa {|}^{s}]<\infty$ and $E[|\lambda {|}^{t}]<\infty$.*

**Proof of Theorem** **8.** To prove the inequality (25), two cases should be discussed. Case 1: Assume that at least one of the two rough random variables κ and λ is equal to zero. It can be easily deduced that:

$$E\left[|\kappa \tau |\right]=E\left[0\right]=0,\;\sqrt[{s}]{E[|\kappa {|}^{s}]}\cdot \sqrt[{t}]{E[|\lambda {|}^{t}]}=0.$$

The inequality (25) holds obviously for this case. Case 2: Assume that the two rough random variables κ and λ are both greater than zero. In this case, we can obtain $E[|\kappa {|}^{s}]>0$, $E[|\lambda {|}^{t}>0$, and E[|κλ|]>0. Let the function $g\left(x,y\right)=\sqrt[{s}]{x}\sqrt[{t}]{y}$ be defined on the first quadrant. Obviously, the function g(x,y) is concave on the definition domain. Thus for any given point $\left(x_{1},y_{1}\right)$ in the first quadrant, there always exits an inequality that:

$$g\left(x,y\right)-g\left(x_{1},y_{1}\right)\leq i\left(x-x_{1}\right)+j\left(y-y_{1}\right),\;\forall x>0,y>0,$$

where *i* and *j* are two real numbers. Assuming that $x_{1}={E[|\kappa |}^{s}]$, $y_{1}={E[|\lambda |}^{t}]$, $x={\left|\kappa \right|}^{s}$, and $y={\left|\lambda \right|}^{t}$, respectively, we have:

$${g(|\kappa |}^{s}{,|\lambda |}^{t}{)-g(E[|\kappa |}^{s}{],E[|\lambda |}^{t}])\leq {i(|\kappa |}^{s}-{E[|\kappa |}^{s}])+{j(|\lambda |}^{t}-{E[|\lambda |}^{t}]).$$

Take the expected value on both sides simultaneously, and then, we have:

$${E[g(|\kappa |}^{s}{,|\lambda |}^{t})]\leq {g(E[|\kappa |}^{s}{],E[|\lambda |}^{t}]).$$

That is,

$$E\left[|\kappa \lambda |\right]\leq \sqrt[{s}]{E[|\kappa {|}^{s}]}\cdot \sqrt[{t}]{E[|\lambda {|}^{t}]}.$$

  ☐

**Theorem** **9.** *(Minkowski inequality) Assume that a probability space is described by (Ω,A,Pr) and κ and λ are two rough random variables. Provided that s is a positive number satisfying 1≤s<∞, then we have:*

(26) $$\sqrt[{s}]{E[|\kappa +\lambda {|}^{s}]}\leq \sqrt[{s}]{E[|\kappa {|}^{s}]}+\sqrt[{s}]{E[|\lambda {|}^{s}]}.$$

**Proof of Theorem** **9.** To prove the inequality (26), two cases should be discussed similarly. Case 1: Assume that at least one of the two rough random variables κ and λ is equal to zero. The inequality (26) holds obviously. Case 2: Assume that the two rough random variables κ and λ are both greater than zero. In this case, we have $E[|\kappa {|}^{s}]>0$, $E[|\lambda {|}^{s}>0$ and E[|κ+λ|]>0. Suppose that the function $g\left(x,y\right)={\left(\sqrt[{s}]{x}+\sqrt[{s}]{y}\right)}^{s}$ is defined on the first quadrant. Obviously, the function g(x,y) is concave on the definition domain. Thus, for any given point $\left(x_{1},y_{1}\right)$ in the first quadrant, there always exits an inequality that:

$$g\left(x,y\right)-g\left(x_{1},y_{1}\right)\leq i\left(x-x_{1}\right)+j\left(y-y_{1}\right),\;\forall x>0,y>0,$$

where *i* and *j* are two real numbers. Assuming that $x_{1}={E[|\kappa |}^{s}]$, $y_{1}={E[|\lambda |}^{s}]$, $x={\left|\kappa \right|}^{s}$ and $y={\left|\lambda \right|}^{s}$, respectively, we have:

$${g(|\kappa |}^{s}{,|\lambda |}^{s}{)-g(E[|\kappa |}^{s}{],E[|\lambda |}^{s}])\leq {i(|\kappa |}^{s}-{E[|\kappa |}^{s}])+{j(|\lambda |}^{s}-{E[|\lambda |}^{s}]).$$

Take the expected value on both sides simultaneously, and then, we get:

$${E[g(|\kappa |}^{s}{,|\lambda |}^{s})]\leq {g(E[|\kappa |}^{s}{],E[|\lambda |}^{s}]).$$

That is,

$$\sqrt[{s}]{E[|\kappa +\lambda {|}^{s}]}\leq \sqrt[{s}]{E[|\kappa {|}^{s}]}+\sqrt[{s}]{E[|\lambda {|}^{s}]}.$$

  ☐

**Theorem** **10.** *(Jensen’s inequality) Assume that a probability space is described by (Ω,A,Pr) and κ is a rough random variable with a finite expected value E[κ]. Provided that the function g is convex and the expected value of g(κ), E[g(κ)], is finite, then we obtain:*

(27) g(E[κ])≤E[g(κ)].

Further, supposing that the function g(x) is equal to ${\left|x\right|}^{s}$ with s≥1, we have ${\left|E\left[\kappa \right]\right|}^{s}\leq {E[|\kappa |}^{s}]$.

**Proof of Theorem** **10.** For any convex function g(x), there always exists a recognized inequality:

g(x)−g(y)≥i·(x−y),

where *i* is a real number. Since the function *g* is convex, substitute the rough random variable κ for *x* and its expected value E[κ] for *y* separately, and we have:

g(κ)−g(E[κ])≥i·(κ−E[κ]).

Taking the expected value on both sides simultaneously, then we have:

E[g(κ)]−g(E[κ])≥i·(E[κ]−E[κ])=0.

  ☐

Holder’s inequality and Minkowski inequality discuss the relationship between the expected value of rough random variables, while Jensen’s inequality depicts the function character of the expected value of a rough random variable. In view of these inequalities, we can directly deduce some other serviceable inequalities regarding convex functions as follows.

**Theorem** **11.** *Assume that a probability space is described by (Ω,A,Pr) and κ and λ are two rough random variables with E[|κ|]<∞ and E[|λ|]<∞. Provided that the function g is convex, then following Theorems 9 and 10, we obtain:*

E[|κ|]+E[|λ|]≥E[|κ+λ|]≥|E[κ+λ]|.

## 4. Critical Values of Rough Random Variables

For the purpose of depicting a rough random variable more profoundly, Liu [17] gave the definition of the optimistic and the pessimistic value of rough random variables. As an extension of Liu’s work, this section further dives into the properties (monotonicity, continuity and others) of critical values.

**Definition** **13.** *(Liu [17]) Assume that a rough random variable is represented by κ and γ,δ are two positive numbers in (0,1]. Then, we have:*

(28) $$\kappa_{\sup}\left(\gamma ,\delta \right)=\sup\left\{t|\mathrm{Ch}\{\kappa \geq t\right\}\left(\gamma \right)\geq \delta \},$$

*where $\kappa_{\sup}\left(\gamma ,\delta \right)$ represents the (γ,δ) optimistic value of κ, and:*

(29) $$\kappa_{\inf}\left(\gamma ,\delta \right)=\inf\left\{t|\mathrm{Ch}\{\kappa \leq t\}(\gamma )\geq \delta \right\},$$

*where $\kappa_{\inf}\left(\gamma ,\delta \right)$ represents the (γ,δ) pessimistic value of κ.*

**Theorem** **12.** *(Liu [17]) Assume that a rough random variable is represented by κ and γ,δ are two positive numbers in (0,1]. Then, we obtain:*

(30) $$\mathrm{Ch}\left\{\kappa \leq \kappa_{\inf}\left(\gamma ,\delta \right)\right\}\left(\gamma \right)\geq \delta ,\;\mathrm{Ch}\left\{\kappa \geq \kappa_{\sup}\left(\gamma ,\delta \right)\right\}\left(\gamma \right)\geq \delta .$$

**Theorem** **13.** Assume that a rough random variable is represented by κ and a is a real number. Then, we obtain:

(i) when a≥0, ${\left(a\kappa \right)}_{\sup}\left(\gamma ,\delta \right)=a\kappa_{\sup}\left(\gamma ,\delta \right)$ and ${\left(a\kappa \right)}_{\inf}\left(\gamma ,\delta \right)=a\kappa_{\inf}\left(\gamma ,\delta \right)$;

(ii) when a<0, ${\left(a\kappa \right)}_{\sup}\left(\gamma ,\delta \right)=a\kappa_{\inf}\left(\gamma ,\delta \right)$ and ${\left(a\kappa \right)}_{\inf}\left(\gamma ,\delta \right)=a\kappa_{\sup}\left(\gamma ,\delta \right)$.

**Proof of Theorem** **13.** (i) When *a* is equal to zero first, Part (i) holds obviously.

Suppose that the number *a* is greater than zero; we have:

$$\begin{array}{cc} {\left(a\kappa \right)}_{\sup}\left(\gamma ,\delta \right)\;\;\;\; & =\sup\left\{t|\mathrm{Ch}\{a\kappa \geq t\right\}\left(\gamma \right)\geq \delta \}\\ & =a\sup\left\{t/a|\mathrm{Ch}\{\kappa \geq t/a\right\}\left(\gamma \right)\geq \delta \}\\ & =a\kappa_{\sup}\left(\gamma ,\delta \right). \end{array}$$

Take the similarly proven process, and then, we have ${\left(a\kappa \right)}_{\inf}\left(\gamma ,\delta \right)=a\kappa_{\inf}\left(\gamma ,\delta \right)$.

(ii) When proving Part (ii), it is equivalent to proving the two inequalities ${\left(-\kappa \right)}_{\sup}\left(\gamma ,\delta \right)=-\kappa_{\inf}\left(\gamma ,\delta \right)$ and ${\left(-\kappa \right)}_{\inf}\left(\gamma ,\delta \right)=-\kappa_{\sup}\left(\gamma ,\delta \right)$. The proving process is as follows:

$$\begin{array}{cc} {\left(-\kappa \right)}_{\sup}\left(\gamma ,\delta \right)\;\;\;\; & =\sup\left\{t|\mathrm{Ch}\{-\kappa \geq t\right\}\left(\gamma \right)\geq \delta \}\\ & =-\inf\left\{-t|\mathrm{Ch}\{\kappa \leq -t\right\}\left(\gamma \right)\geq \delta \}\\ & =-\kappa_{\inf}\left(\gamma ,\delta \right). \end{array}$$

A similar way can be used to prove that ${\left(-\kappa \right)}_{\inf}\left(\gamma ,\delta \right)=-\kappa_{\sup}\left(\gamma ,\delta \right)$.  ☐

**Theorem** **14.** (Monotonicity and continuity) Assume that a rough random variable is represented by κ. Then, we obtain:

(i) When δ takes any value in (0,1], $\kappa_{\sup}\left(\gamma ,\delta \right)$ can be denoted as a function of γ, which satisfies two characteristics, decrease and left-continuity.

(ii) When γ takes any value in (0,1], $\kappa_{\sup}\left(\gamma ,\delta \right)$ can be denoted as a function of δ, which satisfies two characteristics, decrease and left-continuity.

(iii) When δ takes any value in (0,1], $\kappa_{\inf}\left(\gamma ,\delta \right)$ can be denoted as a function of γ, which satisfies two characteristics, increase and left-continuity.

(iiii) When γ takes any value in (0,1], $\kappa_{\inf}\left(\gamma ,\delta \right)$ can be denoted as a function of δ, which satisfies two characteristics, decrease and left-continuity.

**Proof of Theorem** **14.** (i) When δ takes a fixed value in (0,1], the function $g\left(\gamma \right)=\kappa_{\sup}\left(\gamma ,\delta \right)$ is decreasing obviously.

Next, we prove that the function $g\left(\gamma \right)=\kappa_{\sup}\left(\gamma ,\delta \right)$ is continuous from the left. Assume that a positive progression is denoted by {$\gamma_{m}$}, which approaches to γ. Then, $\left\{\kappa_{\sup}\left(\gamma_{m},\delta \right)\right\}$ is increasing distinctly. If the sequence $\left\{\kappa_{\sup}\left(\gamma_{m},\delta \right)\right\}$ approaches to $\kappa_{\sup}\left(\gamma ,\delta \right)$, the continuity from the left is established. If not, we have:

$$\lim_{m\to \infty }\kappa_{\sup}\left(\gamma_{m},\delta \right)>\kappa_{\sup}\left(\gamma ,\delta \right).$$

Assume $h=(\lim_{m\to \infty }\kappa_{\sup}\left(\gamma_{m},\delta \right)+\kappa_{\sup}\left(\gamma ,\delta \right))/2$. It is clear that:

$$\kappa_{\sup}\left(\gamma_{m},\delta \right)>h>\kappa_{\sup}\left(\gamma ,\delta \right)$$

for all *m*. For a fixed positive number ς, there always exists a set $A_{m}$ where $\mathrm{Pr}\left\{A_{m}\right\}\geq \gamma_{m}$ such that:

$$\underset{\tau \in A_{m}}{\inf}\mathrm{Tr}\left\{\kappa \left(\tau \right)\geq h\right\}\geq \delta -\varsigma$$

for each *m*. Define:

$$A^{*}=\overset{\infty }{\underset{m=1}{⋃}}A_{m}.$$

Visibly, $\mathrm{Pr}\left\{A^{*}\right\}\geq \mathrm{Pr}\left\{A_{m}\right\}\geq \gamma_{m}$. Assuming that *m* approaches to ∞, we get $\mathrm{Pr}\left\{A^{*}\right\}\geq \gamma$. Then:

$$\mathrm{Ch}\left\{\kappa \geq h\right\}\left(\gamma \right)=\underset{\mathrm{Pr}\{A\}\geq \gamma }{\sup}\underset{\tau \in A}{\inf}\mathrm{Tr}\left\{\kappa (\tau )\geq h\right\}\geq \underset{\tau \in A^{*}}{\inf}\mathrm{Tr}\left\{\kappa (\tau )\geq h\right\}\geq \delta -\varsigma .$$

Assuming that ς approaches to zero, we have $\mathrm{Ch}\left\{\kappa \geq h\right\}\left(\gamma \right)\geq \delta$. Hence, $h\leq \kappa_{\sup}\left(\gamma ,\delta \right)$. The continuity from the left is established.

(ii) When γ takes a fixed value in (0,1], the function $g\left(\delta \right)=\kappa_{\sup}\left(\gamma ,\delta \right)$ is decreasing obviously.

Next, we prove that the function $g\left(\delta \right)=\kappa_{\sup}\left(\gamma ,\delta \right)$ is continuous from the left. Assume that a positive progression is denoted by {$\delta_{m}$}, which approaches to δ, then $\left\{\kappa_{\sup}\left(\gamma ,\delta_{m}\right)\right\}$ is increasing distinctly. If the sequence $\left\{\kappa_{\sup}\left(\gamma ,\delta_{m}\right)\right\}$ approaches to $\kappa_{\sup}\left(\gamma ,\delta \right)$, the continuity from the left is established. If not, we have:

$$\lim_{m\to \infty }\kappa_{\sup}\left(\gamma ,\delta_{m}\right)>\kappa_{\sup}\left(\gamma ,\delta \right).$$

Assume $h=(\lim_{m\to \infty }\kappa_{\sup}\left(\gamma ,\delta_{m}\right)+\kappa_{\sup}\left(\gamma ,\delta \right))/2$. It is clear that:

$$\kappa_{\sup}\left(\gamma ,\delta_{m}\right)>h>\kappa_{\sup}\left(\gamma ,\delta \right)$$

for all *m*. According to Definition 13, we have:

$$\mathrm{Ch}\left\{\kappa \geq h\right\}\left(\gamma \right)\geq \delta_{m}$$

for each *m*. Assuming that *m* approaches to ∞, we have:

$$\mathrm{Ch}\left\{\kappa \geq h\right\}\left(\gamma \right)\geq \delta .$$

Hence, $h\leq \kappa_{\sup}\left(\gamma ,\delta \right)$. The continuity from the left is established. Taking the similarly proven process, Parts (iii) and (iiii) can be proven immediately.  ☐

## 5. Conclusions

In this study, we first recalled some basic knowledge of random variables, rough variables and rough random variables. Based on this, some inequalities and properties of critical values, generally used in rough set theory and probability theory, are proven for rough random variables to extend and enrich rough random theory. In this paper, the contribution of our work to rough random theory is as follows: (1) The paper proved the well-known probabilistic inequalities including Markov inequality, Chebyshev inequality, Holder’s inequality, Minkowski inequality and Jensen’s inequality for rough random variables. (2) This paper delved into the critical values, and then, some properties of critical values including the continuity, monotonicity, and others, were proven methodically.
